# Adenosinergic Pathway: A Hope in the Immunotherapy of Glioblastoma

**DOI:** 10.3390/cancers13020229

**Published:** 2021-01-10

**Authors:** Ketao Jin, Chunsen Mao, Lin Chen, Lude Wang, Yuyao Liu, Jianlie Yuan

**Affiliations:** 1 Department of Colorectal Surgery, Affiliated Jinhua Hospital, Zhejiang University School of Medicine, No. 365, Renmin Eastern Road, Jinhua 321000, Zhejiang, China; mcs@zju.edu.cn (C.M.); lynne_1121@163.com (L.C.); lyy21918555@zju.edu.cn (Y.L.); 2 Central Laboratory, Affiliated Jinhua Hospital, Zhejiang University School of Medicine, Jinhua 321000, Zhejiang, China; wangruth123@163.com; 3 Department of Neurosurgery, Affiliated Jinhua Hospital, Zhejiang University School of Medicine, No. 365, Renmin Eastern Road, Jinhua 321000, Zhejiang, China

**Keywords:** brain tumor, glioblastoma, immunotherapy, adenosine, CD73, CD39

## Abstract

**Simple Summary:**

Glioblastoma multiforme (GBM) is the most aggressive type of brain tumor with dismal survival and poor response to conventional therapies. Therefore, the development of immunotherapy for GBM treatment is necessary. However, the rigorous immunosuppression in the GBM-microenvironment (GME) is a crucial impediment for GBM immunotherapy. The adenosinergic pathway (AP) is a major player in suppressing antitumor immune responses in the GME. We reviewed the current GBM immunotherapies and elaborated on the role of AP in the immunopathogenesis, treatment, and even prognosis of GBM. Tumor cells metabolize pro-inflammatory ATP to anti-inflammatory adenosine using CD39 and CD73 enzymes. Adenosine suppresses immune responses through the signaling of adenosine receptors on immune cells. The preclinical results targeting AP in the GBM showed promising results in reinvigorating antitumor responses and overriding chemoresistance. We suggest that future clinical studies should consider this pathway in combination therapies along with other immunotherapeutic approaches.

**Abstract:**

Brain tumors comprise different types of malignancies, most of which are originated from glial cells. Glioblastoma multiforme (GBM) is the most aggressive type of brain tumor with a poor response to conventional therapies and dismal survival rates (15 months) despite multimodal therapies. The development of immunotherapeutic strategies seems to be necessary to enhance the overall survival of GBM patients. So far, the immunotherapies applied in GBM had promising results in the primary phases of clinical trials but failed to continue their beneficial effects in later phases. GBM-microenvironment (GME) is a heterogenic and rigorously immunosuppressive milieu wrapping by an impenetrable blood-brain barrier. Hence, in-depth knowledge about the dominant immunosuppressive mechanisms in the GME could foster GBM immunotherapy. Recently, the adenosinergic pathway (AP) is found to be a major player in the suppression of antitumor immune responses in the GME. Tumor cells evolve to metabolize pro-inflammatory ATP to anti-inflammatory adenosine. Adenosine can suppress immune responses through the signaling of adenosine receptors on immune cells. The preclinical results targeting AP in GBM showed promising results in reinvigorating antitumor responses, overriding chemoresistance, and increasing survival. We reviewed the current GBM immunotherapies and elaborated on the role of AP in the immunopathogenesis, treatment, and even prognosis of GBM. We suggest that future clinical studies should consider this pathway in their combination therapies along with other immunotherapeutic approaches.

## 1. Introduction

Brain tumors are heterogeneous tumors that can be classified into two general categories based on their origin. The primary brain tumors stem from the brain, while the origins of metastatic types are other organs that have metastasized to the brain [1,2]. Approximately 80% of brain malignancies originate from glial cells and are called gliomas [3]. According to the 2016 World Health Organization Classification of Tumors of the Central Nervous System, diffused gliomas are categorized into different types, including Astrocytomas, Oligoastrocytomas, Oligodendrogliomas, and Glioblastoma [4]. In this updated classification, molecular parameters are combined with the histological patterns. For instance, the mutation status of isocitrate dehydrogenase (IDH)-1/2 gene and 1p/19q codeletion status are two molecular parameters in classifications of gliomas [4,5,6]. The classification of brain tumors is thoroughly reviewed in [4,5,6]. Glioblastoma multiforme (GBM) is the most malignant and common type of brain tumor in adults. GBM can arise from astrocyte, oligodendrocyte, and even neural stem cells, and therefore, is not classified in a specific category of gliomas [7]. The word multiforme indicates the heterogeneity of this tumor in terms of molecular markers, physiopathology, clinical manifestations, and response to treatment [8].

The average survival in GBM without treatment is three months and with current treatments it is 12–19 months [9,10]. Standard treatment includes surgery, radiotherapy, and chemotherapy [9]. Temozolomide (TMZ) is the gold-standard chemotherapy used in GBM due to its high permeability to the blood–brain barrier (BBB). TMZ is usually given after surgery for six weeks with radiotherapy [11]. Despite these multiple treatments, the recurrence rate of GBM is very high, with 2-year and 5-year survivals of 26.5% and 7%, respectively [10,12]. Steroids are also used to reduce cervical edema [9]. Recently, two other treatments for GBM have been approved in the United States: (I) bevacizumab, a monoclonal antibody (mAb) against vascular endothelial growth factor (VEGF) receptor [13], and (II) tumor-treating fields [14]. However, the effectiveness of both treatments remains controversial. Accelerated approval of bevacizumab in GBM by the FDA indicates the urgent need for advanced and targeted treatment. Due to the ineffectiveness of current treatments on GBM, various types of targeted therapies, such as immunotherapy, raised hopes in the treatment of GBM. Herein, we provide the updates on immunotherapy of GBM with a focus on the role of the adenosinergic pathway (AP), including adenosine, adenosine receptors (ARs), and ectonucleotidases in the immunopathogenesis and treatment of GBM.

## 2. Glioblastoma Immunotherapy

It was initially believed that the central nervous system (CNS) was an immune-privileged organ. Studies on CNS autoimmune diseases such as multiple sclerosis and encephalitis, the discovery of the CNS lymphatic system, and successful treatment of brain metastases, have shown that the CNS has an immunological activity [15]. However, some unique features of the CNS, such as the presence of the BBB, the use of corticosteroids for cerebral edema, and the immunosuppressive mechanisms of brain tumors, caused problems in immunotherapy [16]. Regarding the heterogeneous glioblastoma microenvironment (GME), severe immunosuppression, low mutational burden, and decreased antigen presentation, GBM is very poorly responsive to immunotherapy so far [16] (Table 1). Immune checkpoint inhibitors (ICIs) have become a promising immunotherapy approach in the treatment of many solid tumors (reviewed in [17]). In this method, inhibitory ICs that cause immune exhaustion are blocked, thereby restoring the immune cells’ ability to induce antitumor responses [17,18]. The prerequisite of ICI treatment is the overexpression of ICs in the tumor microenvironment (TME). Overexpression of ICs has been reported only in some subtypes of GBMs [19]. Clinical trials on GBMs have demonstrated that ICIs do not have a significant advantage over other therapies such as bevacizumab, radiotherapy, and chemotherapy. Hence, they proposed a combination of therapies or ICI applications as a neoadjuvant therapy before surgery [20,21,22]. The combined use of several ICIs, although improving the response to treatment, increases their toxicity and the likelihood of CNS autoimmunity [23,24].

In addition to ICIs, the use of mAbs and their derivatives such as nanobodies, single-chain variable fragment (scFv), bispecific T-cell engager (BiTE), and immunotoxins is also a routine method in immunotherapy [29,68]. Bevacizumab was the first mAb to be accelerated and approved in GBM [13]. This anti-VEGF mAb prevents angiogenesis in the TME [13]. Application of mAbs against endothelial growth factor receptor (EGFR) also yielded promising results in initial studies but was discontinued in clinical trials due to a lack of significant increase in patient survival and rising safety concerns [30,31,32]. The EGFR variants, especially EGFR class III variant (EGFRvIII), are overexpressed in a considerable part of GBM patients, making them an ideal target for immunotherapy [69]. However, the association of EGFR overexpression and mutations with the overall survival of patients is still controversial [70]. Moreover, the results of trials showed EGFRvIII downregulation following targeted therapy against EGFRvIII [35,71]. This has raised the question of whether EGFRvIII mutation represents a driver mutation, or maybe it is only a passenger mutation with no considerable impact on the survival of glioma cells. Currently, other generations of conjugated mAb are being studied in trials. The greatest challenge of mAb therapy in brain tumors is the large size of mAbs and the lack of proper penetration into the TME due to the BBB. The smaller derivatives of mAb or making the BBB permeable to these factors could enhance the treatment responses [29].

The application of autologous T cells genetically engineered with a chimeric antigen receptor (CAR) demonstrates remarkable efficiencies in many blood cancers and solid tumors [72]. These cells are against a tumor-specific antigen (TSA) and can sustain antitumor activity with the help of various costimulatory molecules [72]. The CAR T cells used in GBM were against EGFRvIII, interleukin 13 receptor-α2 (IL13Rα2), human epidermal growth factor receptor-2 (HER2), and Eph receptor-A2 (EphA2) [35,36,37,72]. The results of the trials indicate a relative response to this treatment. Given the heterogeneity and high plasticity in the GME, the use of a specific CAR T cell reduces the expression of the target antigen, and the tumor escapes the CAR T cell response [9]. Therefore, studies on the application of bivalent and trivalent CAR T cells are ongoing [37]. Another way to overcome antigen escape is to use BiTEs along with CAR T cells. Choi et al. developed an anti-EGFRvIII CAR T cell, which also expresses anti-EGFR BiTEs [38]. It initially targets positive EGFRvIII cells and then recruits T cells specific for wild-type EGFR to the TME. The initial results against heterogeneous GBMs were promising [38].

Tumor vaccines containing TSAs are another cancer immunotherapy method aiming to stimulate the patient’s adaptive immunity against TSAs [29]. Peptide vaccines containing EGFRvIII and survivin peptides in patients who were positive for these antigens raised proper responses, although the issue of antigen escape in this method is also challenging [40,41,42]. Ex vivo pulsing the patient’s autologous dendritic cells (DCs) with specific peptides (in ICT-107) or tumor lysate (in DCVax) in DC vaccines stimulates a better immune response than peptide vaccines [44,45]. This type of treatment is a personalized treatment that can overcome the high heterogeneity of GBM in patients. However, immunosuppressive GME causes pulsed DCs to become inefficient in antigen presentation. Initial clinical trials of tumor vaccines alone or in combination with bevacizumab or chemotherapy and surgery have yielded encouraging results [9,40,41].

According to initial observations of tumor regression in viral infections, viral therapy is currently used in various cancers, mostly solid tumors [73]. Viruses can be used in gene therapy, delivering the desired genes to the TME. These genes mainly produce pro-apoptotic proteins (in VB-111 vaccine), inflammatory cytokines (in Ad-RTS-hIL-12 vaccine that encodes IL12 conditionally), or enzymes that convert prodrugs to anticancer drugs (in Toca-511) [46,50,51]. Another type of virus therapy involves oncolytic viruses that selectively infect and lyse cancer cells in which antiviral responses are impaired [73]. Adenovirus, herpes simplex virus, and poliovirus are being studied in GBM and have shown a relative response in combination with other treatments [9]. Viral therapy can also stimulate innate and adaptive immune systems that enhance antitumor responses [9].

As can be seen, most of the immunotherapy methods used in GBM have been effective in the preclinical and early clinical stages but have not been very successful in the higher stages of the clinical trials (Table 1). There are several reasons for such an inadequate response in GBM patients. High heterogeneity of GBM between patients and high plasticity, even in one patient at different times, makes GBM resistant to immunotherapy [16]. Evaluation of tumor markers before treatment and development of personalized medicine can lead to overcoming GBM heterogeneity and plasticity. The severe immunosuppressive GME appears to be another barrier to immunotherapy. Immunosuppression in GME undergoes numerous and complex mechanisms so that single-arm immunotherapy cannot break this tolerance. Besides local immune suppression, GBM can suppress systemic immunity in the patient [16,74,75,76]. The GME-infiltrated T cells are mainly differentiated to regulatory T cells (Tregs) due to the high levels of tumor growth factor (TGF)-β and indoleamine-2,3-dioxygenase (IDO) in the GME [77,78]. IDO metabolizes tryptophan to kynurenine, leading to a change in the phenotype of microglial cells (CNS-resident macrophages) or tumor-associated macrophages (TAMs) to an M2 phenotype [67]. M2-TAMs promote tumor progression by further suppressing immune responses and expressing ICs [67].

On the other hand, the use of corticosteroids in GBM to reduce cerebral edema increases immunosuppression and reduces immunotherapy effects [79]. Interestingly, studies have shown that radiotherapy and chemotherapy, such as TMZ in some cases of GBM, can increase immunosuppression and decrease the effects of ICI, which challenges combination therapy [80,81]. Furthermore, the low mutational burden in GBM limits neoantigen production and presentation to the adaptive immune system [82]. All of the mentioned mechanisms make GBM an immunologically cold tumor. Knowing the different aspects of immunosuppression in GBM makes it possible to achieve a successful strategy in GBM immunotherapy by targeting several pathways simultaneously.

## 3. Role of Adenosinergic Pathway in Antitumor Immune Response

ATP inside the cell is a valuable energy source used by all cells. Cellular damage, hypoxia, and nutrient deficiency lead to the active and inactive release of ATP into the extracellular environment [83]. Therefore, ATP outside the cell is considered a damage-associated molecular pattern (DAMP), which binds to the P2X7 receptors on the surface of immune cells, causing the formation of inflammasome and inflammation progression [83,84].

Tumors have evolved to alter the inflammatory mediators to anti-inflammatory ones to evade antitumor immune responses. One of these approaches is the conversion of inflammatory ATP to anti-inflammatory adenosine [85]. The adenosinergic pathway (AP) role in suppressing immune responses was proposed in 1957 when Chu et al. showed that extracellular adenosine suppresses T cell antitumor responses against lymphoma cell lines [86]. Today, the AP, including adenosine, enzymes that produce and metabolize it, and ARs are leading factors in modulating anticancer immune responses. Extracellular ATP is converted to adenosine by cell surface ectonucleotidase enzymes [87]. In this process, the enzyme ectonucleoside triphosphate diphosphohydrolase-1, known as CD39 or NTPDase-1, dephosphorylate ATP to adenosine monophosphate (AMP) [87]. Ectohydrolase and pyrophosphatase enzymes called CD38 and CD203a, respectively, are also able to produce AMP, but their substrates are NAD^+^ and ADP ribose [88]. The enzyme 5′-nucleotidase, known as CD73, hydrolyze the last phosphate from AMP to produce adenosine [87]. Conversion of AMP to adenosine can also be accomplished by membrane phosphatases [89], although the central pathway for adenosine production from ATP is the CD39-CD73 pathway. The adenosine produced has a very short half-life (approximately one second) with one of the following three fates: (I) Re-conversion to ATP by the activity of adenylate kinase (AK) and nucleoside diphosphate kinase (NDPK) inside or outside the cell [90]. (II) To be metabolized to inosine by adenosine deaminase (ADA) and conversion to uric acid [57]. (III) Binding to its receptors, ARs [91]. Under physiological conditions, the production of ATP, AMP, and adenosine is precisely controlled. Though, in pathological conditions such as cancer, the imbalance of these pathways causes the adenosine accumulation outside the cells 100-fold more than its physiological concentration [87]. This sharp increase stimulates the signaling of ARs expressed on the cell surface.

ARs, known as the P1 receptors, are seven-transmembrane G protein-coupled receptors (GPCRs), whose signaling is mediated by adenylate cyclase (AC). The four types of ARs include A1R, A2aR, A2bR, and A3R. A1R has the highest affinity for adenosine, followed by A3R and A2aR [92]. These receptors’ high affinity causes them to be activated even at low concentrations of adenosine [85,92]. A2bR has the lowest adenosine affinity and is activated only at pathological adenosine concentrations [85,92]. A2Rs are paired with Gs protein and activate AC to increase intracellular cyclic AMP (cAMP) levels. Contrarily, the signaling of A1R and A3R is through Gi and Go proteins, which inhibit AC and reduce intracellular cAMP levels [91]. A2bR and A3R also act through Gq and the phospholipase C signaling pathway, which leads to the production of inositol triphosphate (IP3), the release of intracellular calcium, production of diacylglycerol (DAG), and the activation of protein kinase C (PKC) [91]. ARs, especially A2Rs, also activate the signaling pathway of mitogen-activated protein kinase (MAPK), P38 kinase, extracellular signal-regulated protein kinase (ERK)-1,2, and the mammalian target of rapamycin (mTOR) [85]. Regarding their signaling pathways, A2Rs are considered as the main immunomodulator AR [91]. Adenosine signaling through A2Rs on immune cells reduces the secretion of inflammatory mediators, including interferon (IFN)-γ, interleukin (IL)-12, tumor necrosis factor (TNF)-α, perforin, and granzyme [91,93,94]. It also increases anti-inflammatory mediators such as IL-10 and TGF-β, and VEGF, as well as ICs [85,95]. A2R signaling increases differentiation of immunosuppressive cells such as Treg and M2 macrophages [96], while reducing the proliferation and inflammatory activities of T cells, B cells, NK cells, DCs, and innate immune cells such as granulocytes and innate-lymphoid cells (ILCs) [85,93,94,97,98,99,100].

**Figure 1:**
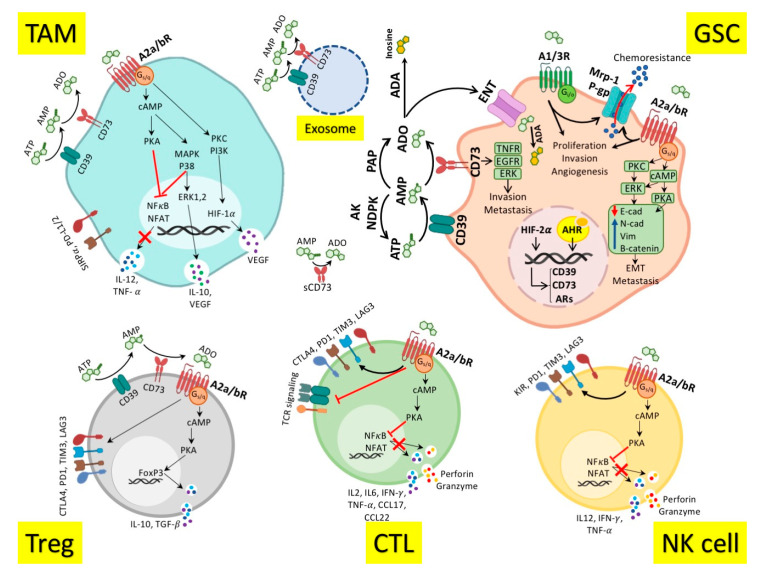
Role of the adenosinergic pathway in the Glioblastoma microenvironment. Adenosine (ADO) is produced from ATP following the enzymatic activity of CD39 and CD73 on the glioblastoma cancer stem cells (GSCs), regulatory T cells (Tregs), tumor-associated macrophages (TAMs), and extracellular vesicles (EVs). Soluble CD73 is also involved in ADO production. ADO binds to the ADO receptors (ARs) on the GSCs and increases the proliferation, invasion, angiogenesis, metastasis, and chemoresistance. The chemoresistance is mediated by the upregulation of Mrp-1 and P-gp that extrude chemtherapeutic agents out of the cells. The GSCs invasion and metastasis are mediated by downregulation of E-cadherin and upregulation of N-cadherin, vimentin, and β-catenin that increase endothelial-mesenchymal transition (EMT). AR signaling (especially A2aR and A2bR) on natural killer (NK) cells and cytotoxic T cells (CTLs) inhibits the antitumor function of these cells by upregulating the immune checkpoints such as CTLA-4, PD-1, LAG-3, and TIM-3, as well as suppressing the release of inflammatory cytokines (IL-12, IFN-γ, TNF-α, and IL-6). The signaling of A2aR and A2bR on the Tregs and TAMs promotes the release of anti-inflammatory cytokines (IL-10, TGF-β) and the upregulation of immune checkpoints, leading to pro-tumor effects. Alternatively, ADO can be metabolized to inosine by extracellular or intracellular adenosine deaminases (ADAs), which is the basis of some therapeutic modalities. ADO. Adenosine; ARs. Adenosine receptors; ADA. Adenosine deaminase; PAP. prostatic acid phosphatase; NDPK. nucleoside diphosphate kinase; AK. Adenylate kinase; cAMP. Cyclic AMP; PKA. Protein kinase A; PKC. Protein kinase C; ERK. Extracellular signal regulated protein kinase; MAPK. Mitogen-activated protein kinase; PI3K. Phosphoinositide 3-kinase; NF𝜅B. Nuclear factor-𝜅B; NFAT. Nuclear factor of activated T-cells; PD-1. Programmed cell-death protein-1; CTLA-4. cytotoxic T-lymphocyte-associated protein-4; LAG-3. Lymphocyte activation gene-3; TIM-3. T-cell immunoglobulin and mucin domain-containing protein-3; SIRPα. Signal regulatory protein-α; PD-L1/2. PD-1 ligand 1/2; KIR. Killer immunoglobulin-like receptor; VEGF. Vascular endothelial growth factor; TNF. Tumor necrosing factor; TGF- β. Tumor growth factor-β; IFN-γ. Interferon-γ; TCR. T cell receptor; Mrp-1. multiple drug-associated protein-1; P-gp. P-glycoprotein; ENT. Equilibrative nucleoside transporter; HIF. Hypoxia-inducible factor; AHR. Aryl-hydrocarbon receptor; Cad. Cadherin; Vim. Vimentin; EMT. Endothelial-mesenchymal transition; sCD73. Soluble CD73.

Tumor cells increase adenosine production and decrease its consumption in the TME by upregulation of CD39 and CD73 and downregulation of AK [101,102,103,104]. On the other hand, A2R overexpression and signaling in the TME suppress antitumor immune responses [91] (Figure 1). Special TME conditions, including hypoxia, high TGF-β levels, and signaling of aryl hydrocarbon receptors (AHRs), trigger the expression of CD39/CD73 and Ars in the TME. Tumor cells, myeloid, and lymphoid immune cells, and even stromal cells and fibroblasts in the TME, express the AP components [85,101,102,103,104]. Due to the loose binding of CD73 via the glycosylphosphatidylinositol (GPI) anchor to the membrane, the soluble form of CD73 is available in the TME and blood of patients. The exosomal forms of CD39/CD73 are also reported in the TME [105,106]. Notably, the endothelial-mesenchymal transition (EMT) process has a reciprocal relationship with adenosine. EMT can increase adenosine, and reciprocally, adenosine signaling promotes EMT and increases metastasis [57,103]. The AP roles in EMT might accentuate the role of adenosine in metastatic brain tumors [107,108]. Adenosine can promote tumor progression in immune-independent ways, as well. It increases cancer cell proliferation, invasion, metastasis, and resistance to treatment via enhancing the stemness feature of cancer cells [57,94,103]. Interestingly, CD73 can also increase tumor invasion and metastasis independently of adenosine by binding to the extracellular matrix as well as activating the TNF receptor pathway and tyrosine kinases such as EGFR and ERK [109,110].

## 4. Adenosinergic Pathway in the Glioblastoma Immunopathogenesis

As mentioned, the severe immunosuppressive properties of the GME have limited the response to immunotherapy. AP is a critical immunosuppressive pathway in glioma and glioblastoma [55] (Table 2). Severe hypoxia in GBM causes ectonucleotidases overexpression and adenosine accumulation in the GME [59]. In a study to find the main factor of immune suppression in the glioma and glioblastoma microenvironment, Ott et al. examined the expression of various immunosuppressive molecules including cytotoxic T-lymphocyte-associated protein-4 (CTLA-4), B-/T-lymphocyte attenuator (BTLA), programmed cell-death protein-1 (PD-1), lymphocyte activation gene-3 (LAG-3), T-cell immunoglobulin and mucin domain-containing protein-3 (TIM-3), T-cell immunoreceptor with immunoglobulin and immunoreceptor tyrosine-based inhibition motif (ITIM) domains (TIGIT), Killer-cell immunoglobulin-like receptor (KIR), CD160, CD73, CD39, and A2aR on the surface of tumor-infiltrating T cells [55]. They showed that the highest expression in both gliomas and glioblastomas was related to A2aR, followed by PD-1 and CD39 [55]. The reason why CD73 is not among the highly expressed molecules is the focus of this team on T cells. The majority of CD73 is located on the surface of tumor cells, while CD39 is mostly expressed on the tumor-infiltrating T cells [62]. The cooperation of tumor-derived CD73 and T cell-derived CD39 produces adenosine [59,62]. CD39/CD73/A2aRs overexpression in the GME and their role in the immune suppression, tumor invasion, and angiogenesis suggest this pathway as an immunosuppressive candidate with high-priority in GME [55,111].

In addition to the T cells, the presence of AP molecules on the surface of macrophages in the GME also plays an essential role in immune suppression [104]. TAMs comprise 20–40% of the total GME cells [112,113,114]. They mostly derived from brain-resident microglial cells or myeloid macrophages that infiltrated into the GME. They can be distinguished from other infiltrating cells through their high expression of CD11b, human leukocyte antigen (HLA)-DR, and CD14 [112]. Within CD11b + HLA-DR + CD14 + TAMs, the pro-inflammatory M1 cells CD192+ and the anti-inflammatory M2 cells are CD163+/CD206+ [112]. These anti-inflammatory M2-TAMs are associated with poor prognosis and resistance to chemotherapy and ICI [115,116,117]. The elevated IDO increases kynurenine production in the GME, which consequently induces CD39 expression on TAMs by activating AHR [67]. In the GME of ICI-resistant patients, there is a group of CD73^hi^ macrophages that persist even after ICI immunotherapy and are involved in immune suppression and ICI-resistance [104]. These myeloid macrophages are recruited from the peripheral blood to the GME and have different genetic signatures from the brain-resident microglial cells [104].

The effects of AP and CD73 in the suppression of GME-infiltrating NK cells have also been observed. It has been shown that CD73 overexpression limits NK cell infiltration into the GME, suppresses their responses, and eventually reduces the survival of GBM patients [59,62].

Studies propose glioblastoma stem-like cells (GSCs) as a chief player in GBM recurrence [58]. The prominent markers of GSCs are prominin-1 (CD133), sex-determining region Y-box 2 (SOX2), CD15, CD44, and A2B5 [118,119,120,121]. However, there is no universal marker to define GSCs. The proportion of GSCs in the GBM is variously based on the method of identification, type and grade of tumors, and the region of sampling. It could vary between less than 1% and higher than 80% of tumor cells and is predominant at the edge of the tumors [118,122,123]. In severe hypoxia, overexpression of hypoxia-inducible factor (HIF)-2α upregulates ectonucleotidases and adenosine production in the GME. Adenosine activates GSCs by stimulating A2bR and A3R, leading to disease progression, angiogenesis, and chemoresistance [124,125] (Figure 1). Adenosine signaling via A3R on GSCs converts them to endothelial cells and increases tumor angiogenesis [56,103]. A2bR and A3R signaling causes infiltration of GSCs to other healthy parts of the brain and increases GBM invasion [57,111]. Moreover, it has been reported that the A3R signaling could upregulate the matrix metalloproteinase-9 (MMP-9), VEGF, and inactivate the pro-apoptotic Bad protein in GBM cells. These changes induce invasion, angiogenesis, and chemoresistance in GBM cells [126,127,128]. Therefore, the A2bR and A3R are also upregulated in the GME and are associated with immunosuppression and tumor progression [55,56,103] (Table 2).

Figure 1 illustrates the role of AP in the GME. CD39 and CD73 are highly expressed on the GSCs, Tregs, TAMs, and extracellular vesicles (EVs) in the GME [129,130]. The tandem ectonucleotidase activities of CD39 and CD73 produce adenosine from ATP [91]. Adenosine binds to ARs on the GSCs and increases the proliferation, invasion, angiogenesis, metastasis, and chemoresistance [55,103]. The chemoresistance is mediated by the upregulation of multidrug resistance protein-1 (Mrp-1) and P-glycoprotein (P-gp) that extrude chemotherapeutic agents out of the cells [58,111,124]. The GSCs invasion and metastasis are mediated by downregulation of E-cadherin and upregulation of N-cadherin, vimentin, and β-catenin that increase EMT [57,103]. AR signaling (especially A2aR and A2bR) on NK cells and cytotoxic T cells (CTLs) inhibits the antitumor function of these cells by upregulating the immune checkpoints and suppressing the release of inflammatory cytokines such as IL-12, IFN-γ, TNF-α, and IL-6 [59,62,85,93,94]. The signaling of A2aR and A2bR on the Tregs and TAMs promotes the release of anti-inflammatory cytokines, including IL-10, and TGF-β, as well as upregulation of immune checkpoints. Therefore, AP has critical roles in restraining antitumor immune responses, leading to GBM progression [29,67,85,104].

## 5. Targeting Adenosinergic Pathway in Glioblastoma Immunotherapy

The immunotherapy failure in GBM might be due to the lack of knowledge about the predominant immunosuppressive agents in the GME and the targeting of less important pathways. For example, it has recently been shown that TIM-3 and LAG-3 do not have more expression in the GME than other checkpoints, whereas they are currently targeted in several clinical trials in GBM patients (NCT03058290, NCT02658981). In this regard, Ott and colleagues showed that the A2aR, CD39, and PD-1 as highly expressed molecules in the GME [55]. However, they showed that the A2aR blockade alone could not restore the antitumor potential of T cells [55]. The failure of A2aR inhibition in the controlling of GBM can have several causes. First, A2a was targeted as monotherapy, and other AP components were not targeted in this study. Blocking A2aR might shift the adenosine signaling to A2bR, another A2Rs whose expression was not evaluated in this study. A2bR expression in the GME is found to be 20 times higher than in healthy brains [111]. The key role of A2bR in GSCs survival and GBM growth in recent studies confirm the importance of targeting this receptor along with A2aR [60,111].

Moreover, the A3R antagonist (MRS1220) reduced tumor growth and decreased angiogenesis in the preclinical models of GBM, indicating the role of A3R in GBM progression and angiogenesis [56]. Hence, more ARs, including A2bR and even A3R, must be targeted to block the AP signaling (Table 2). Interestingly, the A3R blockade also reduces resistance to vincristine chemotherapy [58]. Modulation of ARs such as A1R by an agonist and A2bR by an antagonist increases the GBM sensitivity to TMZ [111,131]. These findings suggest the use of AR inhibitors in combination with chemotherapy.

Given the diversity of ARs, their conflicting roles in various cancers, and the lack of approved AR antagonists in the clinic, reducing adenosine levels in the TME could be an alternative. Niechi et al. investigated recombinant ADA to reduce the adenosine level in the GME [57]. Recombinant ADA is currently prescribed in severe combined immunodeficiency (SCID) disease, making it easier to get approval in the treatment of GBM [132]. Treatment of GSCs with ADA in hypoxic conditions, reduces adenosine levels by 75% in an HIF-2α-dependent pathway, leading to a decrease in chemoresistance, EMT, migration, and invasion of these cells [57].

Another way to lessen the adenosine is by blocking the adenosine producer enzymes CD73 and CD39. In vitro and in vivo inhibition of CD73 using its antagonist APCP leads to GBM regression and activation of GME-infiltrated T cells [62,133]. Considering the role of CD73^hi^ myeloid cells in immunosuppression of the GBM, patients with high levels of these cells are resistant to anti-PD-1 treatment [104]. A GBM mouse model showed that CD73^-/-^ mice had significantly higher survival than CD73^+/+^ ones [104]. Moreover, the use of anti-PD-1 and anti-CTLA-4 antibodies in CD73^-/-^ mice with GBM significantly increased survival compared to the same treatment in wild-type mice with GBM [104]. This finding demonstrates the beneficial effects of CD73 targeting in combination with ICI for GBM immunotherapy. Given the effects of CD73 in reducing NK cell infiltration to the GME and suppressing NK cell responses, studies have suggested using CD73 inhibitors in combination with NK cell therapy in GBM [59]. Besides adenosine decreasing effects, targeting CD73 can inhibit the adenosine-independent immunosuppressive and pro-tumor effects of CD73, such as tumor invasion and metastasis [63,64,133]. Targeting CD73 also improves patients’ response to chemotherapeutic agents such as vincristine [61].

In addition to chemical inhibitors and mAbs, the use of small interfering RNAs (siRNAs) in CD73 inhibition was promising [134,135]. Regarding the better outcomes in the local administration of siRNA, Azambuja et al. used the nasal pathway to block CD73 expression in the CNS. In this approach, siRNA can penetrate the BBB through the olfactory pathway [63,65]. They used cationic nanoemulsion (CNE) to protect siRNA, improve its delivery and distribution in the CNS, and increase its half-life [63]. The in vitro use of CNE-CD73-siRNA inhibited CD73-mediated GBM growth. In the preclinical model, it also reduced tumor volume by 60% without causing toxicity in other organs [63]. In order to find the exact mechanisms underlying the CNE-CD73-siRNA effects on the GBM regression, the immunological effects of CNE-CD73-siRNA in the GME were investigated. They found that this treatment induced tumor cell apoptosis, reduced immunosuppressive cells, such as Treg, TAM, and microglia, and instead, increased inflammatory markers such as IL-6, CCL17, and CCL22 [64]. However, the infiltration of effector CD4^+^ or CD8^+^ T lymphocytes into the GME did not change. Thus, the effects of CD73 inhibition on GBM regression are partly due to altering the GME from immunosuppressive to the inflammatory environment by acting on TAMs and Tregs [64]. CD73 downregulation with CNE-CD73-siRNA also increased TMZ sensitivity even in TMZ-resistant cell lines [66]. Although TMZ itself reduces adenosine, in vivo studies showed that nasal use of CNE-CD73-siRNA had a much greater inhibitory effect on tumor growth than TMZ [66]. This might suggest that inhibition of CD73, besides adenosine depletion, also has adenosine-independent therapeutic effects in GBM. The effect of CNE-CD73-siRNA on GBM regression was so significant that the addition of TMZ could not have more synergistic effects [66].

A noteworthy point regarding CD73 inhibition is that in GBM, and especially the mesenchymal type of GBM, GSC-derived prostatic acid phosphatase (PAP) is also involved in the metabolism of AMP to adenosine [136]. Therefore, in order to achieve better results, all adenosine-producing pathways or signaling should be targeted.

Besides CD73, overexpression of CD39 is also reported in the GME [55,67]. This overexpression that could be even higher than CD73 is induced by AHR on GBM cells and increases the immunosuppressive properties of TAMs [67]. Therefore, studies also suggest CD39 as a target in GBM immunotherapy [55,67]. In this regard, it has been observed that CD39 blocking with ARL67156 improved T cell responses in the GME [62].

In general, in patients with high expression of AP components, targeting these molecules, especially targeting the entire AP, can alter the immunosuppressive GME to the immune-active environment and have outstanding effects in controlling GBM [59,60] (Table 2). It should be noted that the GME suffers from severe complex immunosuppressive mechanisms, such as anti-inflammatory cytokines (TGF-β and IL-10), various ICs, and suppressive immunometabolism. Obviously, monotherapy cannot have dramatic effects on tumor inhibition, and comprehensive multi-arm immunotherapies are required to get the appropriate responses. The encouraging results in the ICI therapy of the CD73^-/-^ mouse model of GBM along with the roles of AP in the NK cells could promise the combination of AP-targeting methods with ICI and NK cell therapy [59,104].

**Table 2:** The roles of adenosinergic pathway components in the prognosis and treatment of GBM.

Target	Pro-Tumor and Immunosuppressive Roles	Diagnostic/Prognostic Roles	Therapeutic Potentials
CD39	- Has the third highest expression among ICs expressed on the GME T cells [55] - Suppresses antitumor immune responses, leading to tumor invasion and angiogenesis [55] - Highly expressed on TAMs and caused resistance to chemotherapy and ICIs [67,115,116,117]	- Overexpression is associated with poor prognosis, resistance to chemotherapy and ICIs [67,115,116,117] - Downregulation is a favorable prognostic factor in DFS [62] - CD39^+^ EVs could be a diagnostic/prognostic factor in GBM [129,130]	- ARL67156 (CD39 antagonist) improves T cell responses in the GME, and regresses the GBM [62]
CD73	- Highly expressed on tumor cells, and T cells, and myeloid macrophages in the GME [55,62,104] - Suppresses antitumor immune responses, leading to tumor invasion and angiogenesis [55,111] - Reduces the response to ICIs, chemokine receptor blockade, and chemotherapy [104,137,138] - Limits NK cells infiltration into the GME and suppresses their function [59,62] - Has adenosine-independent pro-tumor and pro-metastatic roles [63,64,133] - Highly expressed in the TME of mesenchymal-GBM, leading to immune-suppression and treatment-resistance [136,139]	- Overexpression is associated with poor prognosis, and reduced overall survival by 27% [58,59,61,62,104,111,124] - Serves as a prediction factor of treatment response to ICI, chemokine receptor blockade, and chemotherapy [104,137,138] - Downregulation is a favorable prognostic factor in DFS [62] - CD73 overexpression in PBMCs could be diagnostic factor in IDH-1 mutated glioma patients [55] - Prognostic biomarker for overall survival and response to treatment in mesenchymal-GBM patients [59] - CD73^+^ EVs could be a diagnostic/prognostic factor in GBM [129,130]	- APCP (CD73 antagonist) and anti-CD73 mAbs: - Augments the GME-infiltrated T cell responses, regresses GBM and increases survival in preclinical model [62,104,133] - Enhances the response to ICIs such as anti-PD-1 and Anti-CTLA-4 [104], and chemotherapy such as vincristine [61] - Proposed to enhance NK cell therapy in GBM preclinical models [59] - Inhibits also adenosine-independent pro-tumor function of CD73 [63,64,133] - CD73-specific siRNA: - Has high penetration into the BBB, and greater delivery through the nasal administration [63] - Inhibits GBM growth by 60% without significant adverse events [63] - Induces tumor cell apoptosis, increases inflammatory mediators and inhibits GME-infiltrated Tregs and TAMs [64] - Increases temozolomide sensitivity [66]
A1R	- Activates GSCs leading to tumor progression and chemoresistance [60]	- Prognostic factor of tumor progression and chemoresistance, especially resistance to temozolomide [60]	- A1R agonists increase the GBM sensitivity to temozolomide [60]
A2aR	- Has the highest expression among ICs expressed on the GME-infiltrated T cells, and causes immune suppression, tumor invasion and angiogenesis [55]	- Prognostic factor of tumor progression, overall survival, and poor response to immunotherapy [55]	- Anti-A2aR monotherapy could not fully restore antitumor potential of T cells [55]
A2bR	- Has ≥20 times higher expression in the GME compared to the healthy brains, and causes immunosuppression and tumor progression [55,111] - Activates GSCs leading to tumor progression, invasion, and chemoresistance [60,111,124,125]	- Prognostic factor of tumor progression and chemoresistance, especially resistance to temozolomide [55,60,111]	- A2bR antagonists increase the GBM sensitivity to temozolomide [111]
A3R	- Upregulated in the GME with immunosuppressive and pro-tumor effects [55,56,103] - Activates GSCs leading to tumor progression, invasion, and chemoresistance [124,125] - Converts GSCs to endothelial cells and increases tumor angiogenesis [56,103]	- Prognostic factor of tumor progression, chemoresistance, and angiogenesis [56,58,103,124,125]	- MRS1220 (A3R antagonist) reduces tumor growth, angiogenesis, and chemoresistance in GBM preclinical models [56,58]
ADA	- ADA and ENT1 are downregulated in the GME, leading to adenosine accumulation [57,136]		- Recombinant ADA reduces adenosine levels by 75% in GSC culture, leading to decrease in chemoresistance, EMT, migration, and invasion of GSCs [57]
PAP	- GSCs-derived PAP is involved in adenosine production in mesenchymal-GBM [136]		- It is proposed to be targeted along with CD73 and CD39 in mesenchymal-GBM [136]

GBM. Glioblastoma multiforme; ICs. Immune checkpoints; GME. Glioblastoma microenvironment; TAMs. Tumor-associated macrophages; ICIs. Immune checkpoint inhibitors; DFS. Disease-free survival; EVs. Extracellular vesicles; NK cells. Natural killer cells; mAbs. Monoclonal antibodies; TME. Tumor microenvironment; PBMCs. Peripheral-blood mononuclear cells; IDH-1. Isocitrate dehydrogenase-1; PD-1. Programmed cell death protein-1; CTLA-4. Cytotoxic T-lymphocyte-associated protein-4; siRNA. Small interfering RNA; BBB. Blood-brain barrier; Tregs. Regulatory T cells; GSCs. Glioblastoma stem-like cells; ADA. Adenosine deaminase; ENT1. Equilibrative nucleoside transporter-1; PAP. prostatic acid phosphatase.

## 6. The Role of Adenosinergic Pathway in Glioblastoma Prognosis

Overexpression of AP components in GBM and their role in suppressing immune responses can introduce them as a biomarker of prognosis and even response to treatment (Table 2). CD73 and CD39 overexpression has been associated with poor prognosis in GBM [67], so that their downregulation is a favorable prognostic factor in disease-free survival (DFS) [62]. Moreover, CD73 overexpresses in gliomas with isocitrate dehydrogenase-1 (IDH1) mutation, and studies proposed that the CD73 overexpression in peripheral blood mononuclear cells (PBMCs) of glioma patients can be a diagnostic factor for IDH1-mutated glioma in cases where biopsy or surgery are not feasible [55].

In a cohort study of 525 GBM patients, it was found that the genetic signature of CD73^hi^ macrophages in the GME is associated with reduced overall survival (OS) by 27% [104]. These CD73^hi^ myeloid cells cause a diminished T cell infiltration and lack of response to anti-PD-1. Hence, the genetic signature of these cells in GBM patients can be considered a predictor of response to anti-PD-1 treatment [104]. Some clinical trials have targeted chemokine receptors in GBM patients [137,138]. Although these chemokine receptors are highly expressed in GBM, the success of these clinical trials was not considerable [137,138], partly due to the immunosuppressive effects of CD73^hi^ myeloid cells [104]. This indicates the necessity of investigating AP molecules before choosing the type of treatment.

The expression of CD73, A2bR, and A3R in the GME also plays a critical role in chemoresistance that is associated with increased expression of multiple drug-associated protein-1 (Mrp1) and P-glycoprotein (P-gp) in patients with high CD73 and A2bR [58,61,111,124]. A3R activity in chemoresistance is mainly mediated by acting on GSCs [103]. This might indicate the necessity of evaluating the CD73, A2bR, and A3R status in determining chemotherapy response in GBM patients.

Various studies have shown resistance to treatment and poor survival of patients with the mesenchymal type of GBM [139]. In this type, CD73 is highly expressed on the GME while the expression of equilibrative nucleoside transporters (ENTs), which reduces extracellular adenosine, is low. This can result in adenosine accumulation and further immunosuppression in mesenchymal GBM [136]. Interestingly, in a group of mesenchymal GBM patients with low CD73 expression, more prolonged survival was observed. This finding suggests CD73 as an important prognostic biomarker in GBM and especially mesenchymal GBM [59].

Recently, glioma cell-derived EVs have also been shown to express CD39 and CD73 and are able to metabolize ATP to adenosine, which plays a role in antitumor immune suppression and tumor progression [129,130]. These EVs may act as diagnostic and prognostic markers for GBM in the future, which requires further study.

## 7. Conclusions

GBM is the most aggressive type of brain tumor with dismal survival rates and a poor response to conventional therapies. The development of immunotherapeutic modalities seems to be necessary to enhance antitumor treatments. So far, the immunotherapies applied in GBM have had promising results but have failed to continue their beneficial effects in the later phase of clinical trials. High heterogeneity and rigorous immunosuppressive features of the GME necessitate an in-depth knowledge about the dominant immunosuppressive mechanisms in the GME. Recently, AP is found to be a chief player in the suppression of antitumor immune responses in the GME. The preclinical results targeting AP in GBM showed promising results in reinvigorating antitumor response, overriding chemoresistance, and increasing survival. Significantly, most of our knowledge about the role of AP in GBM immunotherapy comes from the preclinical studies that should be confirmed in clinical settings. Future clinical studies should consider this pathway in combination therapies along with other immunotherapeutic approaches.

## Figures and Tables

**Table 1:** Advantages and disadvantages of the current immunotherapies in GBM.

Immunotherapy	Advantage	Disadvantage	Refs.
Immune checkpoint inhibitors (PD-1, CTLA-4, LAG-3, TIM-3, IDO, CD27)	Tolerable Reinvigorate antitumor T cells Promising results in preclinical and first phases of clinical studies Proposed as a neoadjuvant therapy	Grade I-II toxicity in monotherapy Grade III-IV in multi ICI therapy No significant advantage (better OS and PFS) over bevacizumab or TMZ Various IC expression levels in patients Decreased effects in patients receiving TMZ	[19,20,21,22,25,26]
Bevacizumab (anti-VEGF)	FDA-approved for GBM Prevents angiogenesis Has an anti-edema effect	Accelerated approval after phase I/II No outstanding results in extending PFS and OS	[27,28,29]
Cetuximab (anti-EGFR)	Tolerable Promising results in preclinical studies	No significant survival benefit in the phase II trial Insufficient BBB penetration due to the large size	[29,30]
Immunotoxins (mAbs conjugated with bacterial toxin or anti-mitotic agents) (Depatuxizumab mafodotin, Losatuxizumab vedotin, ABBV-221, ABBV-231)	Improved survival in combination with TMZ in the phase II trial ABBV-231 is in the phase I trial	No significant survival benefit in the phase III trial Safety concerns Antigen-escape (downregulation of mAb target) New generations are in the evaluation process	[31,32]
Anti-CSF-1R mAb	Decreases the recruitment of TAMs into the GME Under investigation in the phase I/II trial in combination with ICIs	Might have insufficient BBB penetration due to large size	[33,34]
CAR T cell against IL13Rα2, EGFRvIII, Her-2, EphA2	Appreciable safety profile Considerable infiltration into the GME Significant clinical response	Relapse occurred 2–29 months after treatment Immune-escape through antigen loss Heterogeneity of GME made it difficult to use monoclonal CAR T cell for GBM (only one-third of GBM patients are EGFRvIII+) CAR T cells targeting multiple antigens are needed	[9,35,36,37]
BiTE (against EGFR)	Appreciable safety profile Recruits EGFR-specific T cells in the GME Can override antigen-escape in combination with CAR T cells	Heterogeneity of GME challenges the targeting of a specific antigen in all GBM	[38]
Tumor vaccines using specific peptides (Rindopepimut, survivin) or tumor lysate	Tolerable Low off-target effects Improve OS and PFS (mOS:24 months) Synergistic effect in combination with bevacizumab	Rindopepimut is effective only in EGFRvIII+ patients (30% of all GBM) No survival benefits due to the antigen-escape	[39,40,41,42,43]
DC Vaccines (ICT-107:pulsed with six peptides)(DCVax: pulsed with tumor lysate)	ICT-107: Promising results in the phase II trial DCVax: Improves OS to 24 months Override antigen-escape Personalized medicine	2% serious adverse events in DC vaccines Expensive process of personalized medicine	[44,45]
Viral gene therapy: (Toca-511: Metabolize prodrug (FC) to drug (5-FU)) VB-111: delivers pro-apoptotic proteins Ad-RTS-hIL-12: Conditional delivering of IL-12)	Appreciable safety profile Promising results in early trials with a 22% durable response rate Synergistic effects with ICIs	No survival benefit in the phase III trials	[46,47,48,49,50,51]
Oncolytic virotherapy (Adenovirus, polio-rhinovirus chimera, herpes simplex virus)	Safe intratumoral administration, induces innate and adaptive immune responses Turns immunosuppressive to immune-active TME Promising survival results in early trials	Evaluation in phase II trials as a monotherapy or with ICIs	[52,53,54]
Adenosinergic pathway (ARs, CD39, CD73, ADA)	High expression in all types of GME No antigen escape Turns immunosuppressive GME into immune-active GME Reduces angiogenesis Potentiates other immunotherapies such as ICIs, CAR T cell, and NK cell therapy Synergistic effects with conventional therapies Overrides chemoresistance	Not entered in clinical trials yet mAbs might have insufficient BBB penetration All pathway components should be targeted to get maximum results Not effective as monotherapy and should be used as combination therapy	[55,56,57,58,59,60,61,62,63,64,65,66,67]

PD-1. Programmed cell-death protein-1; CTLA-4. cytotoxic T-lymphocyte-associated protein-4; LAG-3. Lymphocyte activation gene-3; TIM-3. T-cell immunoglobulin and mucin domain-containing protein-3; IDO. Indoleamine-2,3-dioxygenase; ICI. Immune checkpoint inhibitor; OS. Overall survival; TMZ. Temozolomide; VEGF. Vascular endothelial growth factor; GBM. Glioblastoma multiforme; PFS. Progression-free survival; EGFR. Endothelial growth factor receptor; BBB. Blood-brain barrier; mAb. Monoclonal antibody; CSF-1R. Colony stimulating factor-1 receptor; GME. GBM microenvironment; CAR. Chimeric antigen receptor; IL13Rα2. Interleukin-13 receptor α2; Her-2. Human epidermal growth factor receptor-2; BiTE. Bispecific T cell engager; mOS. Mean OS; DC. Dendritic cell; FC. Fluorocytosine; 5-FU.5-Flurouracil; ARs. Adenosine receptors; ADA. Adenosine deaminase.

## Data Availability

Not applicable.
